# Elements of Physiology

**Published:** 1843-01-01

**Authors:** 


ELEMENTS OF PHYSIOLOGY. By *J. Müller*, M.D. &c. &c. Translated from the German, with Notes by *William Baly*, M.D. &c. &c. Part VI., containing *Mind, Generation,* and *Development.* London, Taylor and Walton.

ELEMENTS OF PHYSIOLOGY. By *R. Wagner*, M.D. Translated from the German by *R. Willis*, M.D. Part I. *On Generation.* London, Sherwood & Co. 1841.

PROFESSOR MULLER considers the subject of Generation under two distinct heads, viz. under that of homogeneous or non-sexual generation, and that of sexual generation.

In the *first chapter* is considered the multiplication of organic beings by the process of growth; and first in the case of *plants*. When we compare plants in their fully developed state with the same plants in their earliest condition, we see that during their growth their component organs become multiplied, and that parts, which in the very young plant exist singly or in small number, are in the full-grown plant exceedingly numerous. On a more attentive examination, however, we find that something more than a mere multiplication of the organs of a single individual takes place; and that, in fact, the full-grown plant consists of a system of individuals, or of a multiple of the vegetable organism, which constitutes the plant in its earliest condition; as is proved by the properties which parts of this compound system display. A branch of such a plant, separated from the stem and set in the ground, presents all the characters of the parent organism, and continues to live, nourishing itself and increasing. Nor is this power of multiplying the vital force resident in the germ peculiar to plants. Animals also possess the same property. The young polype developed from the ovum or gemmule of a compound polypiferous animal is at first a single individual, actuated by a single independent will. But in proportion as this young creature grows, it becomes transformed into a multiple system of individuals, like that presented by a full-grown plant, and is then regulated in its movements by many distinct centres of volition.

Thus far our author has considered only those organisms which in their compound state, attained by growth, consist of a system of individuals not merely *potentially* but *actually;* the separate members of the compound system having each its proper structure and independent will.

"We now proceed a step further, and find animals which in form appear perfectly simple, and have only one directing will, but nevertheless are systems of parts endued with individual life, and capable of propagating the form and organization of the species. In these animals the component parts or segments undergo multiplication during the process of growth, and a certain number of them becoming separated, whether spontaneously or artificially, preserve the faculty of individual life. The parts thus separated were for a time subject to the will of the parent animal, and in that respect were mere members of its body; but when about to separate themselves from the rest of the system, they acquire a more intimate relation to each other, become actuated by a distinct will, and have, as it were, their own proper centre of action, even while they form part of the body of the parent worm. At length they become detached, and display free voluntary motion. The young individual thus produced consists at first of few constituent parts or members; grows, however, by the appropriation of new matter, and forms another compound system capable of dividing spontaneously or of being divided into several portions, 'potentially' endowed with individual life. For a time this multiple individual is subject to the influence of a single will, and is only so far a multiple animal as the parts which compose it have the capability of becoming independent individuals, which as yet they are not 'de facto.' Subsequently, the individual parts, though still connected, are actual independent beings."

When a nais has in the process of growth multiplied its organism, the portions capable of separating from the parent animal, and of becoming separate individuals, already have the form of a young nais; there being a repetition in the multiple animal of certain segments which the young and simple animal possesses only in small number. But an animal may in form possess none of the characters of a multiple of individuals, and yet be constituted of a number of parts capable of becoming independent beings; as, for example, the hydra, at the time when it is a simple polype animated by one will and destitute of sprouts. In this state, however, the polype is not a multiple of independent individuals, but it is really a multiple of all that is necessary for the development of a polype; for separated portions of its body grow rapidly, and acquire the form of perfect animals. It has been proved even by experiments, that whether the hydra be divided longitudinally or into transverse ring-shaped segments, or whether portions are merely cut out of their side, the separated fragments are in all cases transformed into perfect polypes. Hence it appears that portions of the body of a polype, the limits of which are undefined, contain, like the leaves of plants, all that is essential to an individual of the species. In this respect the planariæ resemble the polypes. To be sure they never undergo multiplication during growth; so as to form a system of independent beings, each endued with a distinct will, but always remain, with respect to volition, simple animals; but yet when divided into eight or ten separate segments, each of these segments will manifest independent life. Nor yet are we to suppose that the hydræ or planariæ, more than other animals, are deficient in organization, that is, in the possession of organs and tissues. Wherefore, since separated fragments of a planaria or a hydra contain within themselves the power of forming an entire animal, it is evident that this formative power must reside in a mass of different structures, as muscular fibres, nervous fibres, &c., whence it follows, that an aggregate of animal tissues of different physiological properties may be animated with a force quite distinct from the specific endowments of the respective tissues.

"The cause which compels a particular portion of a hydra or planaria to the performance of a subordinate function, is the mutual re-action of its component organic matter with that of the entire organism, which is endowed with a centre of nervous action, or a brain. In this condition the primitive formative force of each part remains latent, and its organisation is subservient to the central influence of the organised polype. As soon, however, as the portion of organised matter of a hydra or planaria ceases to be in contact with the rest of the organism which has a centre of action, it becomes withdrawn from the influence of that centre; and then the tendency to individual organisation is manifested. In the process which ensues, the different tissues already formed within the separated mass probably lose their distinctive characters, and become all transformed into formative matter ('cytoblastema'), and germinal or formative cells; the elementary parts of the tissues of the new animal being subsequently produced by the transformation of those cells. For it is thus that the different tissues of the embryo are developed."

The same, or very nearly the same law, prevails in plants. As long as the leaf is connected with the branch, its faculty of reproducing an entire individual of the same species is kept in a latent condition, in consequence of the mutual re-action of the leaf, as an organ of the plant, with the entire branch or plant itself. But if this mutual re-action is arrested by the dissolution of continuity between the leaf and the stem, the organisation which the former received for a special purpose in the general economy becomes useless, and the latent power of reproduction manifests itself by the formation of a bud, or the germ of a perfect plant.

We now come to the *Second Chapter*, which treats of the

MULTIPLICATIONS OF INDIVIDUALS BY THE DIVISION OF PERFECTLY DEVELOPED ORGANISMS.—FISSIPAROUS GENERATION.

Organic beings in the fully-developed condition being virtually multiples of the germ which produced them, they are capable of multiplication by mere division, without the formation of new germs. This may be the result either of the artificial interruption of contact and organic re-action between the different parts of a body, or of spontaneous division.—

1st. *Artificial fissiparous generation.*—Artificial division produces absolute interruption of continuity between parts which have already undergone perfect organisation, and at the same time are equally endowed with vital force; and it thereby causes the force resident in each part to assume an active state, so as to produce a new organism. Thus polypes may be divided in any direction, and the separated portions will develop new individuals. On the contrary, spontaneous division takes place in certain determinate directions. Multiplication of all plants, and of many of the lower animals, may be effected by artificial division. Branches, twigs, or sprouts, separated from a tree continue to live and keep up the species, when planted or engrafted. Such, however, are not examples of true multiplication by division, effected independently of previously-formed buds, as cuttings of plants are generally provided with fully-formed leaf-buds.

Multiplication by artificial division in animals is most likely to be successful, when the organism consists of a succession of parts of similar structure, the number of which increases during growth. This condition, however, is not indispensably necessary, as in the case of Hydræ and Planariæ multiplication may occur, no matter in which direction the division may be made. There are three modes in which artificial division may be made.

1. *Transverse division.*—This succeeds chiefly when the organism has a linear and parallel development, as in plants and worms (Annelida and Entozoa). 2. *Longitudinal division.*—When Hydræ are divided longitudinally, the cut edges quickly apply themselves one to another, so as again to form a tube. 3rd. *Division in all directions.*—It is chiefly in the inferior plants, as the lichens, and in the hydræ amongst animals, that this sort of division still admits of the reproductive power.

2. *Spontaneous fissiparous generation.*—This generally takes place either transversely or longitudinally, or in both directions at the same time. It is only in animals that this mode takes place to any extent, and hence Ehrenberg employs it to assist in determining, in doubtful cases, whether simple organic beings belonged to the animal or vegetable kingdom. This kind of generation is very common among the infusoria, whilst the higher animals never multiply by spontaneous division. The cause of the spontaneous division is the striving of the organism, rendered virtually a multiple by the process of growth, to concentrate the sway of the organic force upon smaller masses.————The occurrence of spontaneous division in the vegetable kingdom is still a subject of dispute. The only modes of increase in plants, according to Ehrenberg, are elongation and the formation of buds; the cases of apparent division depending merely on the separation of buds or gemmules. Meyer, on the contrary, maintains the frequent occurrence of this mode of increase in the case both of entire plants and of the cells of plants.

Chap. III. contains the Propagation of the Species by Buds.

Our author thus accounts for the formation of buds —

"A portion of the substance of an organised being, which is superfluous for its individual life, separates itself in an undeveloped state of organisation from the organism of that being, so far as to assume a special individuality of life, without, however, losing its organic connexion. From the germ thus produced, the special organisations of the species is subsequently developed in the form of a new individual, which may either retain its organic connexion with the parent trunk, or become detached." A bud originally consists merely of the primary elements of all organisation, namely, cells, and contains even these in proportionally small number. The buds of plants are masses of ordinary vegetable cells. "The ovum is distinguished from the bud not only by the circumstance of sexual influence being necessary for its development, but also by its inability to undergo further evolution while forming part of the parent organism, and by its being insulated from the parent plant by distinct membranes...... Any circumstance which arrests the general growth of the organism at any point, or only interrupts the continuity of the cellular tissue, may determine the formation of buds in plants.

1. *Formation of "Buds," or "Gemmæ," in Plants.*

A. *Buds of the Inferior or Non-vascular Plants.*—These buds of plants of low organisation, consist partly of aggregates of cells, parly of single cells. In the moss and liver-wort tribes, they are of the former kind, whilst in the articulated conferæ and filiform fungi they are single cells.

B. *Buds of the more highly-organised Vascular Plants—Axillary and Terminal Buds.*—These are either formed in the axes of the leaves, or at the extremity of the stalk or stem.

2. *Gemmiparous Generation of Animals.*

In the animal kingdom the propagation by the formation of gemmæ or buds is met with to the greatest extent in the class polypifera, and less frequently in the infusoria. The facts with which we are acquainted, relative to the development of the animal tissues, place it beyond doubt, that the buds developed by animals, like those of plants, are at first mere masses of cells, and that these cells not merely increase in number, but undergo a special arrangement and transformation, so as to produce the different tissues of the body.

*Theory of Non-sexual Generation.*—The production of new organic beings from individuals already existing, may be conceived to take place by either of the two following processes. It may be the result of the entirely new formation of germs by the parent organism; or it may be effected by the mere unfolding or setting free of germs contained within the parent, from the commencement of its existence. The latter view is called the "evolution-theory;" and, according to it, the first created embryos of each species must contain within themselves, as it were, in miniature, all the individuals of that species which shall ever exist. Opposed to this theory is the "Epigenesis," according to which existing organisms do not contain the germs of future individuals, encased within them *ab initio;* but each new germ is an entirely new production of the parent organism. The evolution theory is now completely refuted.

*Sexual Generation.*—Here the germs, though endowed with the power of propagating the distinctive properties of the genus and species, and even of the individual, are incapable of undergoing their destined organization, until acted on by the semen, which itself only propagates the peculiarities of the genus, species and individual only by its influence on the ovum. The semen and ovum are sometimes generated in different individuals, in which case, fecundation is effected by the concurrence of the two sexes. Sometimes both semen and ova are formed in different organs of the same individual, as in hermaphrodites. Hence, dualism of sex does not necessarily involve dualism of the individuals, as sexual generation, as well as multiplication by buds and division, may be effected in a single individual. In hermaphrodite animals fecundation is effected either by the concurrence of two or more individuals or by each individual independently.

"In the former case two individuals either fecundate each other simultaneously, the male organ of each impregnating the female organ of the other, as in many mollusca and worms: or only one individual is impregnated in each act of copulation, the sexual organs being so placed that mutual impregnation is impossible, as in the genus Helluo. In this animal fecundation is effected by the concurrence of two individuals; but while one introduces its penis into the other, it does not itself receive the penis of the latter. In such cases, however, reciprocal impregnation is sometimes effected by the union of several individuals in a series, so that *a* is impregnated by *b*, *b* by *c*, *c* by *d*, and *d* by *e*. The last link of such a series is not fecundated, whilst the first does not fecundate."...... "In those hermaphrodite animals, where fecundation takes place in each independently, it is generally effected by the semen finding its way to the ova within the body of the animal, as in the Radiata, according to Ehrenberg, and in the Distomata, according to Siebold. But in some cases the two kinds of sexual organs are repeated several times in the articulated body of the animal, and then two parts of the same animal may be bent towards each other and act respectively as male and female. It is not rare to find two tapeworms united and impregnating each other reciprocally."

No instance is observed of normal hermaphrodism in the Articulata and Vertebrata. The Infusoria, Radiata, Echinodermata and Annelida, are generally hermaphrodite, as are also the Polypi for the most part. The class Entozoa includes genera devoid of sexes,—hermaphrodites and genera with the sexes in distinct individuals. Some Entozoa, however, namely all the Nematoidea of Rudolphi, have the sexes on distinct individuals. Some worms, also, which do not belong to the class Entozoa, have the sexes distinct, and in that resemble the Nematoid Entozoa. The Planariæ are hermaphrodite, as are all the Annelida. Insecta, Arachnida, Crustacea, and all Vertebrata, have the sexes distinct; and the erroneous assertion that hermaphrodite genera, or genera having only the female sex, exist amongst them, has arisen from the superficial observation of the general similarity of form which the sexual organs sometimes present, for example, in many fishes; or from the comparative rarity of the individuals of one sex, for example, of the males in the genus Apus.

"In these classes, when the sexes are distinct, the individuals may be either male, female, or of no sex, though more correctly speaking, the individuals of the latter kind are unfruitful females, or at least females arrested in their development, since they contain imperfect ovaries. Individuals of this third kind occur in several genera of insects, as Bombus, Apis, and Formica. Those of the beekind are called working-bees. The unfruitful ants are destitute of wings, and are directed by their instinct to protect and nourish the larvæ. The imperfect females or working-bees of the genus Bombus are not indeed incapable of pairing; at least some of those which come out of the larva state in the Spring do, according to Huber, pair with the males of the same generation; but they produce only males. These latter males alone are destined to fecundate the proper or perfect females, the brood of which forms a new colony. Amongst the true bees, Apis mellifica, the working-bees are smaller than the perfect females, though they resemble them in many respects. The working-bees are barren; but they are rendered fruitful if, while in the larva state and very soon after their escape from the egg, they receive a particular kind of food, namely, that destined for the nourishment of the queen-bee; and if they are at the same time placed in a larger cell, they acquire all the properties of the queen-bee. If, however, after having received the food proper for the queen-bee, they are placed in a small cell, they produce only males, and are distinguished from the perfect female by their smaller size. The working-bees, therefore, are females, the ovaries of which, in consequence of the nature of their food during the larva state, have remained undeveloped, and which, at the same time that they have suffered this imperfect evolution, have received special instincts. A swarm of bees consists of about fifteen or twenty thousand working-bees, six or eight hundred males, and a single perfect female."

*Sexual Organs.*—These are divided in animals, into *germ-preparing organs*—the testes and ovaries, which are essential, and always present; *transporting or efferent organs*, the vas deferens and oviduct; and *emitting organs*, penis and vagina, which at the same time serve as implements of sexual intercourse. The essential sexual organs, however, which are universally present, are the formative organ, the testis or ovary, and the efferent apparatus, the vas deferens, or oviduct. The sexual organs of both sexes present two perfectly distinct types, characterised by the relation which the formative and efferent organs bear to each other. In one type the efferent organ has the place of a true efferent duct, its walls being continuous with those of the cavities of the formative organ; in the other, the two essential parts of the sexual organs are wholly unconnected, and the ovum or semen first makes its way through the parietes of the formative organ into the abdominal cavity, and escapes thence by a special canal. The product of the formative organ may in this case either fall free into the abdominal cavity before making its way out by the efferent canal, or it may be at once received from the ovary or testis into the open end of the tube which is in the neighbourhood. The male sexual organs of all invertebrata, and of by far the greater number of the vertebrata, are formed after the first type; such is their form in man, mammalia, birds, reptiles, amphibia, and most fishes. The female sexual organs are less frequently formed after this type. The male sexual are seldom formed in accordance with the second type, in which the efferent duct opens directly out of the abdominal cavity, and has no communication with the testis. They never have this form in the invertebrata, and among the vertebrata, only in some few fishes. The female sexual organs are rarely found after the second type among the invertebrata; whilst in vertebrate animals, on the contrary, this type is the prevailing one; the only exception to it being in osseous fishes.

"The simplest form of the female efferent organ, when it is distinct from the ovary, is found in the Cyclostomata, in eels, the Cobitis tænia, and in the Salmonidæ; in which fishes it is a simple opening leading from the abdominal cavity to the exterior......... In the sharks and rays, reptiles, amphibia, birds and mammalia, the short efferent canal of the Cyclostomata is extended into a long tube,—the oviduct. The end of this tube, which opens into the abdominal cavity, sometimes lies close to the ovary, as in man, mammalia, and birds." (*Müller* 461.)

"The general consideration of the morphology of the human organs of generation, show us certain relations which serve to elucidate many physiological phenomena. What we learn, admits of application, and funishes us with analogies in reference to the same system through the whole of the series embraced by the animal kingdom. The *testicle* is composed of *tubuli seminiferi,* canals of great tenuity, which are everywhere copiously surrounded by blood-vessels. The tubuli anastomose with one another in loops. When unravelled from the tangled skein, which they form naturally, they constitute a canal of the delicacy and diameter of a sewing thread of more than a thousand feet in length; the amount of secreting surface is consequently very considerable. The tubuli unite into a variable number of excretory ducts, which pass into a thicker convoluted and looped tube, the *epididymis,* which in its turn terminates in a simple contorted canal, the *vas deferens,* which at its terminal extremity is provided with a diverticular appendage, the *vesicula seminalis.* This last appendage seems to serve partly as a reservoir for the spermatic fluid, partly as an organ of secretion; it is not found in many mammalia. The *prostate* and *Cowper's glands* are secernent organs, the clear viscid secretion of which consists of a transparent liquid intermixed with flocculi and granules which are mostly either normal or altered epithelial cells. The *penis* is a highly vascular organ, copiously supplied with nerves which, in virtue of a peculiar and not yet perfectly understood mechanism of its blood-vessels, receives upon occasion such an afflux of blood, that it enlarges and stiffens, or undergoes erection, by which it is fitted to penetrate into the female vagina. The mucous or internal coat of the seminal vessels is furnished with a flattened cylindrical epithelium, which is continued into the vas deferens; the epithelial cells of the vesiculæ seminales, which are tesselated or united in the manner of a pavement, contain nuclei of considerable size, and, farther, a quantity of granular matter."

"The *ovaria* of the human female have a very compact and solid stroma; each ovarium contains about fifteen fully-formed Graafian vesicles, in which the very small ovula (measuring from the 20th to the 30th of a line) are imbedded in the manner already described; the Fallopian tubes, as well as the uterus, contain muscular fibres, which have the histological (structural) character of those of involuntary muscles; the *vagina* is narrow at its orifice in the virgin, and partially closed by the *hymen,* which, however, leaves an opening of about half an inch in width superiorly; the *clitoris* is small; the mucous follicles situate between the labia and in the vagina secrete a fluid of a peculiar greasy odour. The mucous membrane between the labia pudendi, and covering the hymen and vagina to the middle of the cervix uteri, is covered with a tesselate epithelium; from the point indicated this is replaced by a cylindrate epithelium, which is continued through the tubes to the edges of their fimbriated extremities, where it passes into the tesselate epithelium of the peritoneum; the epithelial cylinders have similar nuclei, and carry cilia the $\frac{1}{300}$th of a line in length. In the mammalia, where the structures are all essentially the same as in man, the cilia may be seen many hours after death in rapid motion. After every menstrual period, certainly after every conception, the ciliate epithelium is thrown off and reproduced; the epithelium of the vagina is always in a state of copious desquamation. The ciliate epithelium is wanting before puberty, and after the period of child-bearing is past; in animals it only makes its appearance when they have attained the age at which they can engender." (*Wagner,* 59.)

### PHÆNOMENOLOGY OF THE GENERATIVE ACT.

*Encounter of the Generative Elements.*—Certain questions require to be solved before a closer insight into the nature of the generative process can be obtained. The chief of these is—Must the generative elements which are severally prepared in the male and female sexual organs come into actual contact to produce their peculiar effects? and the answer being in the affirmative, how is this contact accomplished? In order to solve this, it becomes necessary to turn to the remoter circles of organic nature. In the vegetable kingdom the occurrence of an intimate material contact between the pollen granule with its fovilla, and the nucleus of the ovum, has been demonstrated; it is only when this contact is accomplished, that an embryo is evolved. Other facts and observations go to confirm this same matter. With respect to what takes place in the interior of the female organs of generation in the higher orders of vertebrate animals. Direct experiment has now proved the presence of spermatozoa in the interior of the female sexual organs after the access of the male. Spermatozoa may be found in the recently dead bodies of almost all animals that are frequently in heat, and produce several litters in the course of the year.

Dr. Bischoff, of Heidelberg, has found spermatozoa alive and active in the vagina, in the uterus, through the whole length of the tubes and between their fimbriæ, and finally in the sac or capsule which the peritoneum forms around the ovary, in a bitch after an encounter with the male.

The necessity of immediate contact of the spermatic fluid with the ova has been further proved by experiments on artificial impregnation. The same thing is proved by experiments of an opposite kind, in which the male sperm is prevented from reaching the ova: thus the ova of the frog and of fishes, unless brought into contact with the sperma of their several males, remain unfruitful.

In some of the lower vertebrata the ova of the female are extruded before fecundation has taken place; therefore the separation of the ova from the place of their formation is not connected with their fecundation. In such cases the extrusion may be considered a consequence of the excitement arising from sexual intercourse. Among mammalia and man, however, the rule seems to be, that no ova are detached from the Graafian vesicles unless fecundation has taken place. Hence the improbability of the opinion according to which the product of the ovary, even in cases where fecundation is accomplished internally, is cast loose to meet the sperm. The grounds for believing that the fecundation of the ovum takes place in the ovary are as follows: 1st. The fact of the semen penetrating to the ovary. 2. The circumstance that the ova of birds, and also of mammalia, advance to maturity partially and in succession, and are also detached from the ovaria in the same manner, and that the fecundated ova in different families of the mammalia first make their appearance in the uterus after the lapse of a certain interval of time from the last visit of the male, an interval often so great as to render it improbable that the semen continues accumulated in the uterus during its course. In rabbits, ova are usually found in the cornua of the uterus on the third day after effective coitus; in the dog, and it would appear in the human subject also, the ova do not enter the uterus before the eighth day. 3. The occurrence of cases of extra-uterine conception, which are by no means infrequent either in the human subject or among animals, speak loudly for the impregnation of the ova in the ovary. 4. If the oviducts, or Fallopian tubes be tied or divided in animals immediately after sexual intercourse, no development of ova follows.

In man and the mammalia penetration of the female organs of generation by the male organs is not necessary to impregnation; it is enough that the male sperm be simply so thrown or introduced into the female organs, as that it may by possibility reach the os uteri. In the case of man there are numerous cases which go to prove that fruitful intercourse may take place without actual intromission; thus, men with malformation of the penis,—with hypospadias and epispadias of the worst kind; men who have had the penis partially amputated, in whom but a very imperfect coitus could take place, have all proved themselves capable of engendering.

With respect to the conditions necessary for fecundation as regards the quantitative and qualitative relations of the sperma to the ovum, and the reciprocal influence of these upon one another, the principal are—1st, that the ovum possess a certain maturity of its elements, especially of its yelk. 2. The sperma must be recent. 3. The quantity of the sperma seems to have but little influence on its fecundating power. 4. The sperma must contain spermatozoa.

*Phenomena accompanying the Generative Act.*

Besides the essential phenomena, of impregnation, there is a series of accompaning phenomena, reflections, as it were, in other organic systems, which play a secondary part in the generative act. To this series may be referred those occurrences which have their ground in a participation of the nervous system, and which accompany, in an especial manner, the sexual act. The nerves of common sensation appear to be always in a state of high excitement during the sexual act; an intense feeling of enjoyment is experienced, which reaches its height at the instant of the emission or ejaculation; this ejaculation is itself a reflex action, excited by irritation of the sensitive nerves of the penis. It is the result of two movements; namely, of the persistent contraction of the organic muscular fibres of the vesiculæ seminales, and of the repeated periodical contraction of the animal muscular fibres of the ejaculator seminis and other perineal muscles. In saying that the act of emission is a reflex action, it is understood that the sensory nerves of the glans penis transmit a peculiar sensation to the brain and spinal cord, and these reflect the impression upon the corresponding muscular parts of the genital system: the scrotum is drawn up tight round the testicles, the vesiculæ seminales and prostate are compressed by the levatores ani, and the spasmodic rhythmical contractions of the perineal muscles, particularly the bulbo-cavernosi, effect the vigorous ejaculation of the spermatic fluid. This occurs only during the complete erection of the penis, by which it is fitted to penetrate, more or less, into the vagina.

In the two sexes, the act of coition is attended with pleasureable sensations, but their respective share in the act is different. In the female, the sense of pleasure seems chiefly to be excited by friction of the labia externa and clitoris, which are in a state of turgescence or erection; this excitement, as in the male, often reaches such a degree of intensity as to induce a kind of syncope. The impression made is also reflected on the nervous parts of the internal organs of generation, one effect of which is probably dilatation of the os uteri for the reception of the spermatic fluid. In the female, however, there is no expenditure of nervous power, no energetic rhythmical muscular contractions, when the venereal excitement has reached its height, and no emission of semen; but merely an increased secretion of mucus from the mucous follicles of the vagina, excited by the impressions on the sensitive nerves of the female sexual organs, and serving to lubricate the passage. The man feels exhausted after the act; the woman does not. Whence it may be inferred that the sensitive excitement of the woman is neither so rapidly rendered intense, nor so rapidly depressed, as that of the man: this conclusion accords with the fact, that women bear frequent sexual intercourse better than men, and are much less frequently injured by sexual excess.

*Immediate Consequence of Sexual Intercourse and Impregnation.*

The most immediate consequence of the sexual act, in oviparous animals is the separation of the ova from the ovarium; among some of the lower animals this happens even during the act; in other instances it occurs later, as among insects, birds, mammalia and man. The stimulus of the intercourse is felt by the ovary; in the ripe ova, fecundated or fit for fecundation, the germinal vesicle disappears.

How this happens, whether from rupture or other causes, is not easily determined. It occurs, or may occur, however, before positive fecundation, or contact of the semen has taken place. In the mammalia the separation of the ova from the ovary seems to be dependent on the act of impregnation. It has, is it true, been stated, that cicatrices of the ovaries, resulting from the escape of ova, have been seen in the bodies of virgins; this, however, is not an ordinary occurrence. Generally, it is only after a successful union of the sexes, that one or more Graafian vesicles are found turgid. At a later period, after coition has taken place, the turgid and vascular vesicle of De Graaf bursts, and the ovulum is received into the Fallopian tube. These results of a fruitful union of the sexes, the change in the condition of the ovary, the dehiscence of the Graafian follicle and escape of the ovulum, are now known to be consequences of the direct action of the semen on the ovary itself.

The way in which the ova are transferred from the ovary to the Fallopian tube or oviduct is not very accurately known. In mammalia and birds the proximity of the ovary to the infundibulum of the Fallopian tube must, to a certain extent, facilitate the entrance of the ovum into the latter; but even here there is a phenomenon, as yet unexplained, in the Fallopian tube applying its infundibulum, or fimbriæ, at the time of impregnation, not merely to the ovary, but exactly to that part of it at which there is an ovicapsule on the point of bursting. It is still more difficult to explain the passage of the ova into the Fallopian tube in those animals in which the infundibulum of the tube lies at a considerable distance from the ovary, as in the amphibia, and sharks and rays.

The changes which precede the expulsion of the ovum from its capsule and which the capsule afterwards undergoes, are the following: Both in oviparous animals and in mammalia the posterior part of the capsule swells before the escape of the ovum; but in mammalia this tumefaction is much greater than in other animals, and the swollen capsule appears very vascular, and is filled with a brownish fluid, even before the ovulum has left its cavity. In consequence of this change the contents of the capsule are protruded against its outer wall, which has become thinner, and now projects with the ovulum beneath it in a hemispherical form above the surface of the ovary and thickened follicle. An aperture, the stigma, is then formed at the most prominent point. Immediately after the escape of the ovulum, the cavity of the follicle, or Graafian vesicle, appears very small; in a short time it becomes quite filled with a granular mass and a kind of wart is developed in the site of the former opening. This disappears and the altered follicle remains with the form of the round corpus luteum.

The semen may be supposed to effect fecundation in two ways; either by acting indirectly on the ovum through the medium of the organism, or by acting directly on the ovum. It is now proved that fecundation, following the union of the sexes, results from the direct action of the semen upon the ovum. It is equally certain that fecundation does not depend on any influence of the entire male organism, but on the semen alone. Hence the essential cause of fecundation, wherever it takes place, is not any influence of the male organism on the female, but the action of the semen itself on the female germ.———The part which the spermatozoa play in the fecundating process is not altogether very clear. Do they serve merely to increase the sphere of action of the fertilizing matter, as insects, carrying the pollen, aid in the fecundation of plants, or do they themselves contain the essential fertilizing principle? We would refer the reader to Dr. Barry's ingenious observations on this interesting point of physiology.

*Superfœcundation or Superfœtation.*—With respect to man and the mammalia with fruitful coitus the generative act is closed, and its end accomplished. The rule is, that as soon as ova have been cast loose, the heat ceases in female animals: bitches will not then suffer the approach of the male. In the human female, however, the sexual disposition appears rather to increase during the first weeks after conception has taken place. If one or a first fruitful connexion be followed shortly afterwards, and *before the formation of the decidua,* by a second, in some rare instances *superfœcundation* may ensue. The only cases of superfœcundation that can be relied on, are those in which a woman has had connexion with two men of different races shortly after one another, and has brought twins into the world, a circumstance of which several instances are on record. Negresses have produced a mulatto and a negro at a birth: they had connexion with a European and a negro shortly after one another. Once the decidua is formed and the ovum has reached the uterus, fruitful intercourse is no longer possible, and the cases of *superfœtation* which have been admitted under these circumstances are physiological impossibilities. The cases described as cases of superfœtation are all referrible to twin conceptions, in which one of the fœtuses has perished at some anterior period of the pregnancy, and has been more or less perfectly preserved.

We shall here close our analysis of and extracts from these two valuable contributions to medical science. Professor Müller's work has already received the impress of approbation in preceding Numbers of this Journal —it is, in fact, the most complete work we now possess on the subject; we have not had, until the present occasion, an opportunity of noticing Wagner's work; from what we have as yet seen of it, we consider it an excellent book. From the very great simplicity of its arrangement and the clearness of its style, we are much inclined to think that it will be the favorite, as well with the student as with the practitioner. Dr. Willis already so favorably known to the profession for several excellent original works on practical subjects, more especially for his valuable work on diseases of the urinary organs, has now an additional claim on his professional brethren, both for his elegant translation of Wagner's work, as also for the excellent annotations which he has added to it.

